# Quantifying Pseudosymmetry in Molecular Crystals

**DOI:** 10.1063/4.0000855

**Published:** 2025-10-27

**Authors:** Inbal Tuvi-Arad

**Affiliations:** The Open University of Israel, Raanana, Israel, Israel

## Abstract

Crystal structures that exhibit approximate higher-order symmetry compared with their assigned space group are commonly termed pseudosymmetric. Unlike errors in space group assignments, which are corrected and well-documented in crystallographic databases, information about approximate symmetry is not always readily accessible, and generally lack clear quantification. Here we propose a shift in perspective on crystal pseudosymmetry, introducing quantification through the continuous symmetry measure (CSM) approach. This method, which has proven effective in numerous studies on individual molecules is applied to address crystals in which symmetry arises from the spatial arrangement of molecular sets, irrespective of the symmetry of the individual molecules. The method enables the analysis of extensive crystallographic datasets, uncovering hidden insights associated with approximate symmetry. This approach revises the binary classification of symmetric and asymmetric crystals, introducing a continuous, quantitative, and informative gradient of symmetry. Results of CSM analysis for a variety of crystals will be discussed.

